# Supplementary material for: The Toxic Masculinity Scale: Development and Initial Validation
Source: Behav Sci (Basel). 2024 Nov 14;14(11):1096. doi: 10.3390/bs14111096 (PMC11591014; doi:10.3390/bs14111096)
Supplement: Supplementary file 1 [file behavsci-14-01096-s001.zip › behavsci-3300723-supplementary.pdf]

1. If I cry, I am weak
2. Crying means I am weak
3. Expressing sadness publicly makes me weak
4. Public displays of sadness means I am not masculine
5. I want to dominate others
6. Dominating others is a key to being successful
7. Dominant men are attractive to their partners
8. People are attracted to men who dominate others
9. Muscles are indicators of masculinity
10. Men who are not physically fit aren't masculine
11. Men who aren't involved in contact sports (e.g., football, rugby) aren't real men
12. As a man, it is important to work out
13. As a man, it is important to lift weights
14. Men who wear women's (e.g., dresses) clothing aren't masculine
15. Men who wear dresses/skirts aren't masculine
16. Men who wear makeup aren't masculine
17. Being nice to your partner isn't masculine
18. Publicly displaying affection to/with your partner isn't masculine
19. Posting your partner on social media isn't masculine
20. There are toys specifically for boys
21. The majority of household chores are intended for women
22. Men and women aren't equal
23. Men are superior to women
24. Women are designed to support men
25. Gender and sex are the same thing
26. There are only two genders
27. It is important that men and women are distinct from each other
28. Gender is not different from sex
29. It is propaganda that there are more than two genders
30. A promiscuous man is masculine
31. A man with a lot of sexual partners is more masculine
32. Men cheating on their partner is natural
33. Men are not designed to be monogamous (i.e., have one partner at a time)
34. Sensitive men are weak
35. Men should not be sensitive
36. Men are ready to have sexual intercourse at a younger age than women
37. External factors do not impact my emotions
38. I don't let me emotions dictate my actions
39. Women are more emotional than men
40. Women should not be leaders
41. Men should not work for women
42. My opinion as a man is more important than that of a woman's
43. I don't value the opinion of women
44. A woman's ideas are not as important as a man's
45. I feel comfortable being honest about my emotions with others
46. The more masculine I appear to be, the better I feel about myself
47. Men should enjoy outdoor activities
48. It is more natural for a man to have a job working with his hands

49. Men should use only masculine pronouns (e.g., he/him)
50. I expect men to be tough
51. I don't respect men who cry in public
52. I seek the approval of other men in my life
53. Men don't need the approval of women
54. Men being aggressive is natural
55. Gay men cannot be masculine
56. Men can't rape women because consent isn't a real thing
57. Men's behavior isn't their fault because they are men
58. It is normal for men to use profanity
59. Women should not display masculine behavior
60. I am competitive
61. I don't like to lose
62. When I compete with friends, I always take it seriously
63. Losing is unacceptable
64. Losing is a sign of weakness
65. I am always right
66. I don't lose arguments
67. When I am angry, I lash out
68. I respond to verbal altercations with physicality
69. Fear is unacceptable
70. It is not okay to be afraid
71. When I am afraid, I ignore it
72. I am never scared
73. If I am sad, I bury my feelings
74. I don't share my feelings with my partner
75. *I avoid being honest with my partner*
76. Lying to my partner is okay, because I am a man
77. If I am in pain, I don't let people know
78. I ignore pain when I feel it
79. If I am sick, I refuse to go to the doctor
80. If I don't feel well, I just ignore it
81. I don't let other people direct my actions
82. I don't listen to others
83. On average, men are smarter than women
84. Men are more capable than women
85. Men talking over women isn't rude
86. It is natural for men to be aggressive
87. Aggression is normal for men
88. My opinion is more important than that of others
89. I know more than the typical person
90. I feel angry when someone refuses to fulfill my sexual needs
91. I see it as a challenge when someone refuses my sexual advances
92. Once someone gives their consent, they can't take it back
93. I feel insecure around bigger men
94. I think women dress for male attention
95. Women should dress a certain way
96. Sex workers have no self-respect

97. It’s a woman’s job to take care of the kids
98. Sex work isn’t a career
99. A woman's value is based on her attractiveness
100. A man's value is based on his power and strength
101. Success is a mindset
102. Unsuccessful people are not trying enough
103. There is no such thing as male privilege
104. Men don’t have inherent advantages in life
105. A deeper voice is more masculine
106. When men succeed it is because of hard work
107. Successful women use their bodies to achieve their success
108. Taller men are more masculine
